# Delayed-Onset White Matter Lesions on Brain MRI in Recurrent Non-cerebral Plasmodium falciparum Imported Malaria Without Neurological Symptoms

**DOI:** 10.7759/cureus.76416

**Published:** 2024-12-26

**Authors:** Daniela S Rico, Aengela Jihyoun Kim, Yael Zoken, Suman Radhakrishna, Antonio K Liu

**Affiliations:** 1 Neurology, Ross University School of Medicine, Bridgetown, BRB; 2 Internal Medicine, Adventist Health White Memorial, Los Angeles, USA; 3 Infectious Disease, California Hospital Medical Center, Los Angeles, USA; 4 Neurology, Adventist Health White Memorial, Los Angeles, USA; 5 Neurology, Loma Linda University School of Medicine, Loma Linda, USA

**Keywords:** delayed-onset white matter lesions, imported malaria, malaria brain mri, non-cerebral malaria, recurrent malaria

## Abstract

*Plasmodium falciparum* malaria affects millions of people in certain regions of the world, with neurological involvement and/or cerebral malaria as potential manifestations. Brain magnetic resonance imaging (MRI) abnormalities have been well-documented in cerebral malaria. However, MRI abnormalities in non-cerebral malaria, especially in neurologically asymptomatic patients, are not well understood and have been less frequently reported, especially in non-endemic regions. Additionally, there are no known studies that observe and analyze the presence and progression of these radiological abnormalities over long periods.

Here, we present the case of a patient with recurrent non-cerebral imported malaria infections spanning two decades. Despite a normal brain MRI three years prior, the patient was found to have extensive subcortical white matter fluid-attenuated inversion recovery abnormalities on MRI. This case highlights the possibility that even neurologically subclinical malaria infections can result in significant, long-standing brain changes, raising important questions about the pathophysiology of malaria’s effects on the brain, the potential for cumulative neurological damage over time, and the clinical significance of such findings. In addition, the significance of the location of these lesions in non-cerebral cases remains unclear, particularly in terms of their clinical implications and reversibility. Our findings suggest the need for further studies to evaluate the long-term consequences of malaria infections on the brain, particularly in non-cerebral cases, and to explore whether these radiological abnormalities are reversible or lead to lasting neurological impairment.

## Introduction

Malaria infections present a significant healthcare challenge, especially within endemic areas such as sub-Saharan Africa, South and Southeast Asia, the Middle East, Latin America, and the Western Pacific. The Centers for Disease Control and Prevention (CDC) and the World Health Organization (WHO) classify malaria as a life-threatening disease caused by parasites of the genus *Plasmodium*, transmitted to humans via infected female *Anopheles* mosquitoes. The most threatening species are *Plasmodium falciparum* and *Plasmodium vivax*. In 2020, the WHO reported an estimated 241 million malaria cases and 627,000 malaria deaths globally [1]. The United States is not an endemic area and, therefore, malaria may be less well understood in the United States. However, 68 cases of imported malaria were reported in the United States in 2023 [2]. In the context of the United States, imported malaria is defined as malaria that is acquired outside of the United States, with travel to an endemic region within two years of diagnosis [3].

Uncomplicated malaria is characterized by non-specific findings such as fever, chills, headaches, myalgia, abdominal pain, diarrhea, and cough [4]. Complicated malaria can present with more severe symptoms and outcomes, including anemia, respiratory distress, and multi-organ failure, sometimes leading to death. Neurological manifestations of malaria, often associated with *Plasmodium falciparum*, include seizures, psychosis, agitation, altered extrapyramidal signs, cerebellar ataxia, impaired consciousness, and coma [5]. The WHO defines cerebral malaria as a condition where the patient has at least one hour of coma after the resolution of a seizure or hypoglycemia, with asexual forms of *Plasmodium falciparum* on peripheral blood smears, and no better explanation for the coma [6]. It is also defined by a Glasgow Coma Scale (GCS) score below 11 [7]. Many clinicians would make a diagnosis of cerebral malaria in patients with any neurological manifestation. The mortality rate of cerebral malaria ranges from 10% to 20%, with some sources citing rates as high as 30%. Additionally, up to 25% of children with cerebral malaria experience long-term motor, cognitive, and/or behavioral impairments, with 10% developing epilepsy [6,8].

Magnetic resonance imaging (MRI) studies in cases of cerebral malaria have shown lesions in various brain regions, including white matter, the corpus callosum, brainstem, and cerebellum, as well as subcortical infarcts, cytotoxic edema, and diffuse micro-hemorrhagic changes in the corpus callosum, basal ganglia, and both superficial and deep white matter [4,9,10]. Few studies have reported MRI findings in non-cerebral malaria [11]. The clinical significance of abnormal MRI brain findings in non-cerebral malaria is not well understood. Additionally, there is no known study that tracks MRI abnormalities over long periods in cases of non-cerebral malaria, especially among cases of imported malaria.

## Case presentation

**Figure 1:**
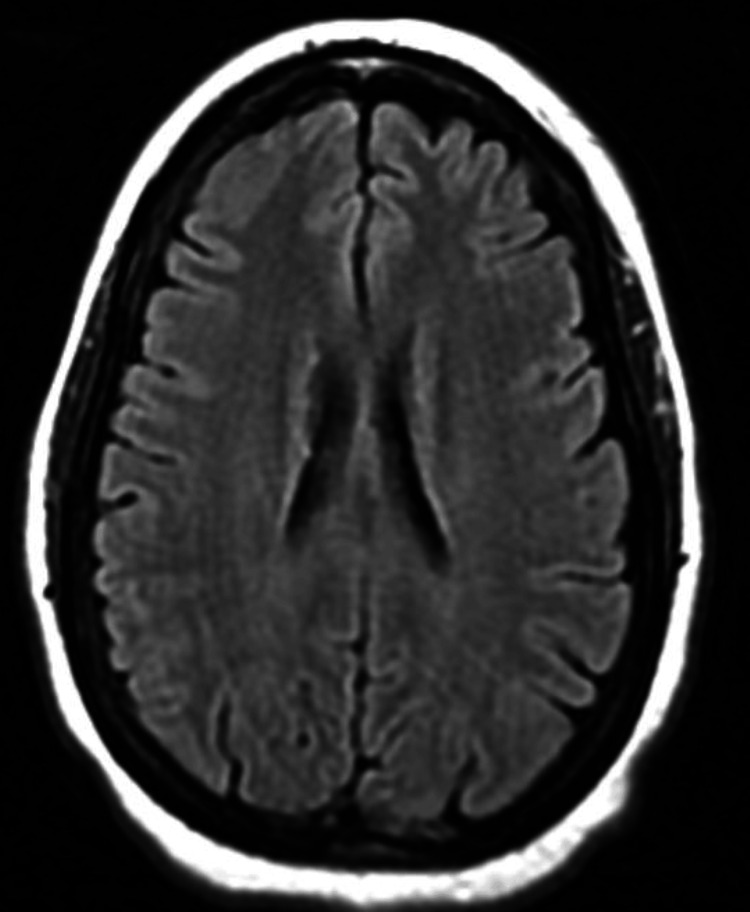
Normal fluid-attenuated inversion recovery sequence of brain magnetic resonance imaging in 2021 (three years prior).

A 50-year-old man presented to the Emergency Department (ED) in 2024 with fever, malaise, and severe headaches for three to four days, following his return from Jamaica two weeks prior. He reported that these symptoms felt very similar to his previous malaria infection. He had a confirmed non-cerebral malaria infection in 2006, during which he had no neurological manifestations. He had no other significant past medical history. The patient, a businessman, traveled frequently between Los Angeles and Jamaica. In 2021, he had an ED visit for headaches, during which a brain MRI was negative (Figure 1).

**Figure 2:**
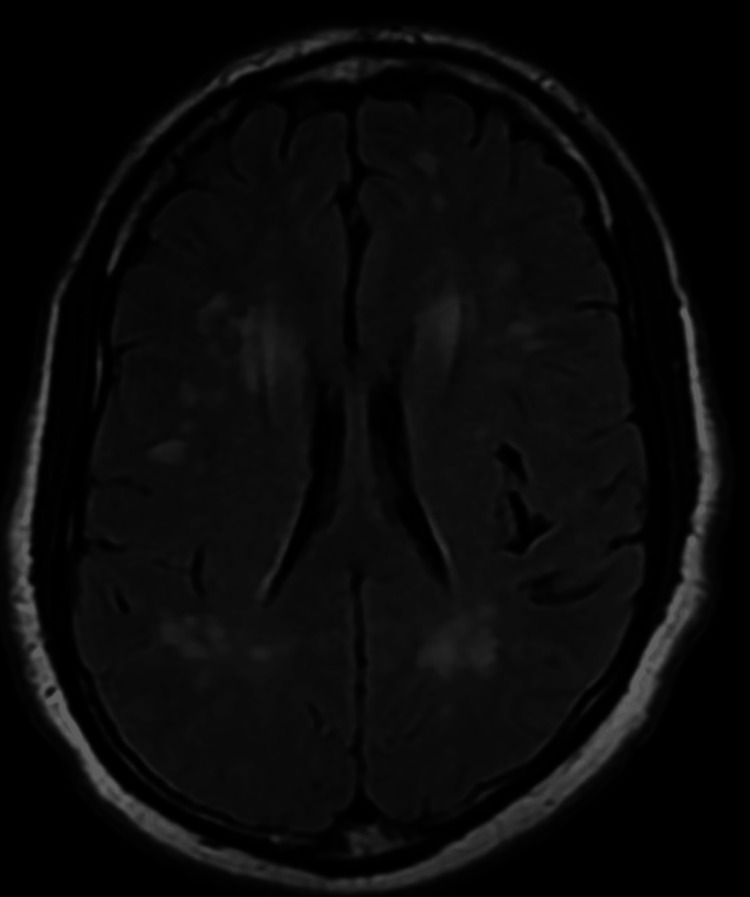
Magnetic resonance imaging fluid-attenuated inversion recovery sequence showing periventricular white matter lesions and deep white matter lesions at the level of the lateral ventricles. They are punctate, bilateral, and diffuse.

**Figure 3:**
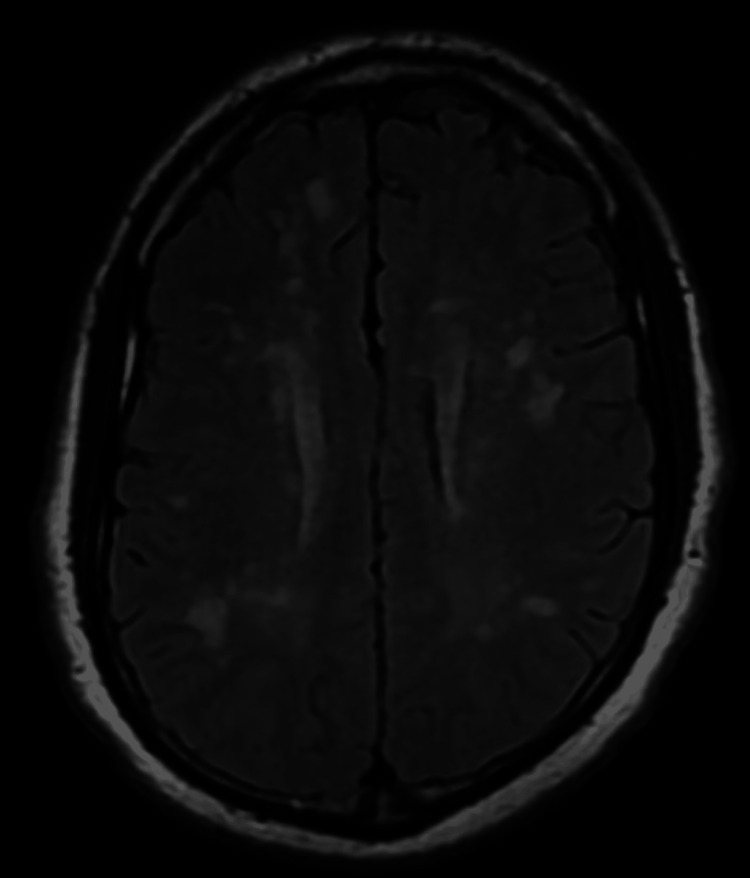
Magnetic resonance imaging fluid-attenuated inversion recovery sequence showing bilateral, diffuse, and punctate periventricular and deep white matter lesions right above the lateral ventricles.

**Figure 4:**
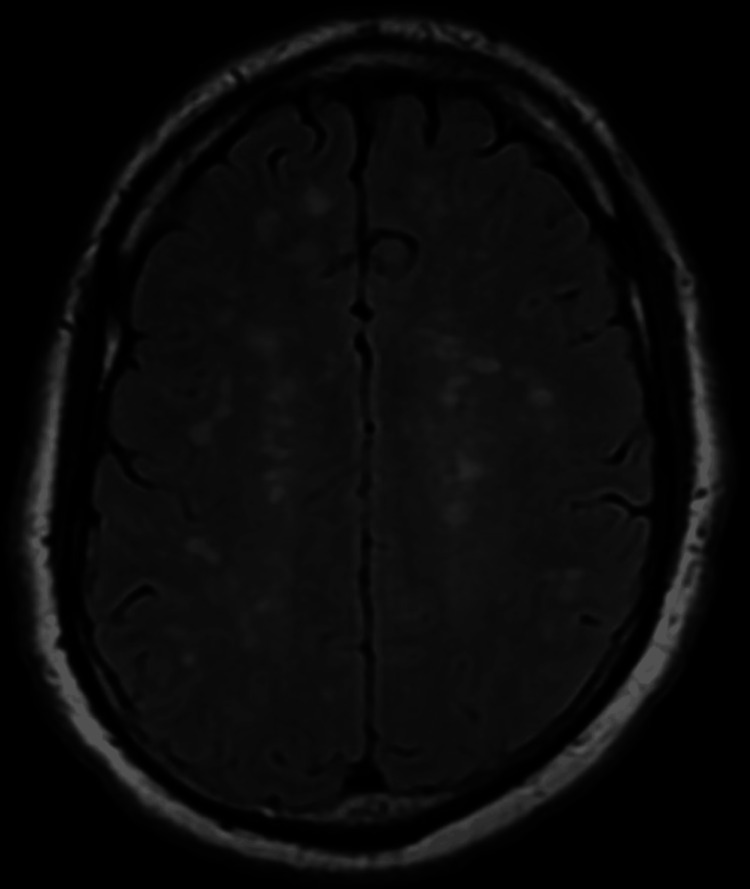
Magnetic resonance imaging fluid-attenuated inversion recovery sequence showing more diffuse white matter lesions.

Upon examination in the ED, the patient was alert, oriented, coherent, and cooperative, with a GCS score of 15. He had a fever of 38.5°C, but other vital signs were within normal limits. The patient exhibited mild meningismus and photophobia. His cranial nerves were intact, and he had normal tone, bulk, strength, sensation, and cerebellar function. His gait was normal. MRI of the brain revealed no diffusion-weighted imaging (DWI) abnormalities but showed significant subcortical white matter lesions on the fluid-attenuated inversion recovery (FLAIR) sequence (Figures 2-4). There was no T1 signal abnormality, and contrast was not administered.

Laboratory results showed white blood cell (WBC) counts between 3,700 and 4,900/µL and platelet counts as low as 58,000/µL, which improved to 119,000/µL. Electrolytes, renal function, and liver function tests remained normal throughout admission. Cerebrospinal fluid analysis revealed 5 WBCs/mm³, 32 mg/dL of protein, and normal myelin basic protein levels at 2.34 ng/mL. Treatment was initiated with atovaquone-proguanil (250 mg-100 mg, four tablets daily). *Plasmodium falciparum* was detected on malarial stains for three days. Parasitemia was 0.2% on day one, <0.1% on day three, and undetectable by day four (Table 1). The patient’s headaches, fever, and malaise resolved rapidly within the first three days of treatment. His neurological examination remained non-focal and unremarkable. He was discharged after six days in the hospital. At a six-week post-discharge follow-up via phone, the patient was asymptomatic and had resumed work. However, he declined a repeat MRI.

**Table 1:** Pertinent laboratory results. CSF: cerebrospinal fluid; WBC: white blood cell

Test	Laboratory value	Reference range
Serum WBC	3,700–4,900 /µL	5,000–11,000 /µL
Platelet	58,000–119,000/µL	140,000–400,000 /µL
CSF WBC	5 WBC/mm^3^	0–5 WBC/mm^3^
CSF protein	32 mg/dL	15–45 mg/dL
CSF myelin basic protein	2.34 ng/mL	<4 ng/mL
*Plasmodium falciparum* day one	0.2%	0
*Plasmodium falciparum* day three	<0.1%	0
*Plasmodium falciparum* day four	0	0

## Discussion

Cerebral malaria has been associated with brain MRI lesions in various regions of the brain. However, studies have also documented brain lesions in patients with non-cerebral malaria, even in the absence of neurological symptoms. Several reports have highlighted the corpus callosum, particularly the splenium, as a common site of lesions in both non-cerebral [9,11-14] and cerebral malaria cases [9,10,14]. However, this location is more frequently spotted in cases of cerebral malaria [12]. One study examining patients with both uncomplicated and severe non-cerebral malaria reported a variety of MRI abnormalities, with the basal ganglia being a common region affected in adults [15]. The basal ganglia may be particularly vulnerable due to its high metabolic demand and limited collateral blood supply, making it more susceptible to hypoxia [16]. In other cases, reductions in apparent diffusion coefficient in the basal ganglia have been observed in cerebral malaria, paralleling findings seen in global hypoxic-ischemic injury [17]. These studies suggest that hypoxic injury to the brain may be a key mechanism underlying MRI abnormalities in malaria, regardless of whether clinical manifestations are present.

In some cases, there have been reports of complete resolution of brain lesions on MRI, despite lingering long-term neurocognitive deficits. For example, one case of cerebral malaria with a positive *Plasmodium falciparum* stain presented with confusion. An MRI on day four revealed diffusion restriction in the white matter and corpus callosum. By day 21, the diffusion restriction had completely resolved on MRI, with improvement in symptoms, but with persistent long-term neurocognitive deficits [18].

In the previous example, the white matter and corpus callosum lesions reversed, yet long-term cognitive and neurological effects remained. While reversible splenial lesions have been reported in both cerebral and non-cerebral malaria, there are no reports citing the reversibility of lesions in the deep white matter [13,14]. Another study found that splenial lesions reversed after 36 days, though without clinical improvement [14]. In cases of non-cerebral malaria, cytotoxic edema on MRI has been shown to reverse within 72 hours in some patients [15]. These findings suggest that neurological symptoms can occur in malaria patients without visible MRI lesions, while MRI lesions can also exist without accompanying neurological symptoms.

The pathology of cerebral malaria is often attributed to sequestered red blood cells adhering to the endothelium of cerebral microvasculature, which can impair blood flow and lead to hypoxic injury [19]. Additional mechanisms may include endothelial cell hypertrophy, fibrin necrosis, microvascular thrombosis, myelin loss, and blood-brain barrier leakage [20]. Brain MRI abnormalities in cerebral malaria may also be related to multi-organ failure, rather than cerebral malaria alone [21]. Cytotoxic edema, resulting from hypoxia due to compromised brain microcirculation in severe malaria, can be observed in both complicated non-cerebral and cerebral malaria [6]. Cytotoxic edema often develops initially in hypoxia-sensitive regions, followed by vasogenic edema during the subacute stage. Depending on the severity of the cytotoxic edema, it may either reverse completely or progress to vasogenic edema [18].

Post-malaria neurological syndrome (PMNS) should be considered in patients who develop neurological symptoms within two months of recovery from acute malaria, where blood smears are negative for malaria parasites and other etiologies have been ruled out. Although the exact mechanism of PMNS is unclear, one proposed explanation is molecular mimicry, where antibodies generated against the malaria parasite cross-react with autoantigens, leading to neurological symptoms [22-24]. Another theory suggests structural brain damage resulting from a recent malaria infection [23] but most evidence supports an immunological process as the primary mechanism behind PMNS [25].

Patients with PMNS often show non-specific changes on brain imaging, affecting both gray and white matter. In 43% of PMNS cases, MRI findings include non-specific hyperintensities, sometimes with enhancement, in subcortical and periventricular areas, the internal capsule, corpus callosum, thalamus, brainstem, and cerebellum [23,24].

A case study of a patient who had contracted malaria two months earlier while in Ghana presented with a seizure in Qatar. Although the patient had no other neurological symptoms, brain imaging (CT and MRI) revealed vasogenic edema in the white matter of the left frontal and right posterior parasagittal regions, suggesting PMNS [20]. In another case of PMNS following uncomplicated malaria, the patient later experienced seizures and MRI findings showed T2 FLAIR hyperintensities in the frontal and occipital lobes. The patient had no other neurological deficits, and after treatment, symptoms resolved. At the time of the follow-up MRI, the brain imaging appeared normal [26].

The clinical significance of MRI brain lesions in both cerebral and non-neurologically significant malaria remains uncertain. Are these lesions clinically significant, and, if so, why are they present in some patients and not others? Could factors such as repeated infections or post-infection immunological responses contribute to the development of these lesions?

Our patient, who had a history of non-cerebral malaria with no neurological manifestations in 2006, presented in 2024 with MRI findings of white matter lesions on FLAIR, despite having a normal brain MRI in 2021. Given the patient’s frequent travel, it is possible that additional malaria episodes occurred between 2006 and 2024, contributing to these FLAIR abnormalities. Alternatively, these lesions may have developed over time as a result of the 2006 infection or multiple exposures to malaria. These findings could be directly related to the malaria infection itself or could represent a post-infection immunological response, such as PMNS, or a combination of both. Of course, it is also possible that these MRI abnormalities could be due to non-malaria-related causes, though this remains uncertain.

As the patient declined further imaging, we cannot determine whether these lesions are permanent or reversible. Most of the existing literature captures cases of non-cerebral and non-neurological malaria at a single point in time or over the short term, with very few studies following patients with long-term imaging. Future studies should focus on long-term follow-up with serial MRIs in patients with both cerebral and non-cerebral malaria to better understand how *Plasmodium falciparum* affects the brain and determine how much time and insult it takes for clinical symptoms to manifest, if ever. Such long-term studies could also provide insights into whether and when brain lesions from malaria are reversible or irreversible, and whether the reversibility of these lesions correlates with clinical improvement.

## Conclusions

This case highlights the potential for significant brain MRI abnormalities in patients with recurrent non-cerebral malaria, even in the absence of neurological symptoms. The development of extensive FLAIR white matter lesions on MRI, despite a normal scan just three years prior, raises important questions about the long-term effects of repeated malaria infections. While the exact mechanism behind these findings remains unclear, the possibility of malaria exposure(s) leading to delayed or cumulative neurological insult should be further investigated. Additionally, the role of post-infection immunological responses, such as PMNS, warrants further exploration in cases with similar imaging abnormalities.

Future studies focusing on long-term follow-up with serial brain MRIs, both in patients with and without clinical neurological manifestations, can be important in understanding the pathophysiology of malaria-induced brain lesions. Such research could provide valuable insights into whether these MRI findings correlate with clinical outcomes and whether they are reversible over time.
